# Impact of post-sepsis cardiovascular complications on mortality in sepsis survivors: a population-based study

**DOI:** 10.1186/s13054-019-2579-2

**Published:** 2019-09-02

**Authors:** Meng-Huan Wu, Po-Yang Tsou, Yu-Hsun Wang, Meng-tse Gabriel Lee, Christin Chih Ting Chao, Wan-Chien Lee, Si-Huei Lee, Jiun-Ruey Hu, Jiunn-Yih Wu, Shy-Shin Chang, Chien-Chang Lee

**Affiliations:** 1 Department of Emergency Medicine, Chang Gung Memorial Hospital, Kaohsiung, Taiwan and Chang Gung University College of Medicine, Taoyuan, Taiwan; 2 Department of Epidemiology, Bloomberg School of Public Health, Johns Hopkins University, Baltimore, MD USA; 3 Department of Emergency Medicine, National Taiwan University Hospital, No.7, Chung Shan S. Rd., Zhongzheng Dist., Taipei, 100 Taiwan; 4 Canberra Hospital, ACT Health, Canberra, Australian Capital Territory Australia; 5 Department of Rehabilitation and Physical Medicine, Taipei Veterans General Hospital, Taipei, Taiwan; 6 Department of Medicine, College of Medicine, National Yang Ming University, Taipei, Taiwan; 7 Department of Emergency Medicine, Chang Gung Memorial Hospital, Keelung, Taiwan and Chang Gung University College of Medicine, Taoyuan, Taiwan; 8 Department of Family Medicine, Taipei Medical University Hospital and School of Medicine, Taipei Medical University, Taipei, Taiwan; 9 Department of Emergency Medicine, Health Data Science Research Group, National Taiwan University Hospital, No.7, Chung Shan S. Rd., Zhongzheng Dist., Taipei, 100 Taiwan

**Keywords:** Survival analysis, Post-sepsis myocardial infarction, Post-sepsis stroke

## Abstract

**Background:**

It remains unclear whether sepsis-related cardiovascular complications have an adverse impact on survival independent of pre-existing comorbidities. To investigate the survival impact of post-sepsis cardiovascular complications among sepsis survivors, we conducted a population-based study using the National Health Insurance Database of Taiwan.

**Methods:**

We identified sepsis patients from the National Health Insurance Research Database of Taiwan using ICD-9-CM codes involving infection and organ dysfunction between 2000 and 2011. Post-sepsis incident myocardial infarction (MI) and stroke were ascertained by ICD-9-CM codes and antiplatelet treatment. We constructed a non-sepsis comparison cohort using propensity score matching to ascertain the association between sepsis and cardiovascular complications. Furthermore, we compared the 180-day mortality and 365-day mortality between patients surviving sepsis with or without post-sepsis MI or stroke within 70 days of hospital discharge. We constructed Cox regression models adjusting for pre-existing comorbidities to evaluate the independent survival impact of post-sepsis MI or stroke among sepsis survivors.

**Results:**

We identified 42,316 patients hospitalized for sepsis, from which we matched 42,151 patients 1:1 with 42,151 patients hospitalized without sepsis. Compared to patients hospitalized without sepsis, patients hospitalized with sepsis had an increased risk of MI or stroke (adjusted odds ratio 1.72, 95% CI 1.60–1.85). Among 42,316 patients hospitalized for sepsis, 486 (1.15%) patients developed incident stroke and 108 (0.26%) developed incident MI within 70 days of hospital discharge. Compared to sepsis survivors without cardiovascular complications, sepsis survivors with incident MI or stroke had a higher mortality rate at 180 days (11.68% vs. 4.44%, *P* = 0.003) and at 365 days (16.75% vs. 7.11%, *P* = 0.005). Adjusting for age, sex, and comorbidities, post-sepsis MI or stroke was independently associated with increased 180-day (adjusted hazard ratio [HR] 2.16, 95% CI 1.69–2.76) and 365-day (adjusted HR 1.90, 95% CI 1.54–2.32) mortality.

**Conclusions:**

Compared to sepsis patients without incident MI or stroke, sepsis patients with incident MI or stroke following hospital discharge had an increased risk of mortality for up to 365 days of follow-up. This increased risk cannot be explained by pre-sepsis comorbidities.

**Electronic supplementary material:**

The online version of this article (10.1186/s13054-019-2579-2) contains supplementary material, which is available to authorized users.

## Background

Sepsis, which refers to life-threatening organ dysfunction caused by a dysregulated host response to infection, is a major public health concern [1]. In Taiwan, the incidence of sepsis has steadily risen from 638 per 100,000 persons to 772 per 100,000 persons from 2002 to 2012, while the annual mortality has steadily decreased from 27.8 to 22.8% over the same time period [2]. While short-term sepsis mortality is decreasing, mid- to long-term sepsis mortality has remained high as many patients die in the subsequent months. Recent studies have suggested that the increased risk of mid- to long-term mortality after sepsis cannot be explained by the pre-existing comorbid conditions before sepsis and might be attributed to increased post-sepsis cardiovascular complications instead [3]. Sepsis increases the risks of cardiovascular complications during and shortly after admission, with up to fourfold increase in the risk [4–7]. The increased risk of cardiovascular complications has been attributed to a variety of pathophysiologic mechanisms, including immunoparalysis, depression of ventricular function, arrhythmia, organ ischemia related to increased oxygen demand, procoagulant changes in the blood, impaired cardiovascular autonomic response, and accelerated atherosclerosis [8–10]. Consistent with prior literature, our previous work using the National Health Insurance Research Database of Taiwan demonstrated that patients with sepsis are at markedly elevated risk of incident MI/stroke during the first 70 days after hospital discharge [11]. We found that after this critical 70-day period, sepsis survivors have a comparable risk of post-sepsis MI/stroke with non-sepsis control patients.

Despite the established association between sepsis and cardiovascular complications, limited information is known about the survival impact of these post-sepsis cardiovascular complications. Among the difficulties in assessing the survival impact of post-sepsis cardiovascular complications are the rarity of post-sepsis cardiovascular complications and the complex competing risk relationship between mortality and post-sepsis incident cardiovascular complications [12, 13]. The aim of this study was twofold. First, we sought to determine the independent association of sepsis with incident cardiovascular disease after sepsis hospitalization with the construction of a non-sepsis comparison group. Second, we sought to ascertain if the increase in mortality associated with cardiovascular events in sepsis patients exceeded the mortality associated with cardiovascular events in non-sepsis patients. The use of a national population-based database allows us to build a large sepsis cohort with a sufficient number of events to compare the outcome between patients with and without incident MI/stroke after sepsis [14, 15].

## Methods

### Data source

The present study used the National Health Insurance Research Database (NHIRD) of Taiwan. Taiwan’s National Health Insurance (NHI) program is a single-payer mandatory health insurance system that covers over 99% of the 23,000,000 people residing in Taiwan. The database used a systematic sampling strategy to select one million participants who were representative of the demographic and geographic region distribution of Taiwan in 2001. This sample, hereafter referred to as the one million longitudinal sample, was followed from 2001 to 2012 to form a closed cohort for research purposes. The database collects outpatient and inpatient electronic records on demographics, eligibility, vital status, diagnoses (International Classification of Diseases, ninth revision, Clinical Modification [ICD-9-CM]), operations, and prescriptions. All claims can be linked in chronological order to provide a temporal sequence of all health services utilization. Patient consent was not required for this study as this was an anonymized electronic database study. This study was approved by the Institutional Review Board of National Taiwan University Hospital. This observational study was performed in accordance with the Strengthening the Reporting of Observational Studies in Epidemiology (STROBE) guidelines for reporting observational studies [16].

### Design and study participants

The study was a population-based cohort study, consisting of all emergency department (ED) or hospital-treated sepsis patients between 2001 and 2011.

### Identification of sepsis cases

Sepsis patients were identified based on the coding system used by Angus et al., which is considered to most closely resemble the latest Sepsis-3 definition among the published algorithms [17]. Operationally, sepsis patients were identified using ICD-9-CM codes for the presence of either a bacterial or fungal infection (Additional file 4: Appendix 1) plus dysfunction of one or more organ systems (Additional file 4: Appendix 2) [1]. The ICD-9-CM codes for the identification of infections in this study were the same as those used by Angus et al. (including 1286 distinct infection codes). The ICD-9-CM codes for acute organ dysfunctions were supplemented with NHI procedure codes to increase specificity. In this manuscript, MI refers to myocardial infarction alone, stroke refers to cerebrovascular events alone, and MI/stroke stands for the combined outcome of MI and stroke.

### Construction of the non-sepsis comparison cohort

**Fig. 1:**
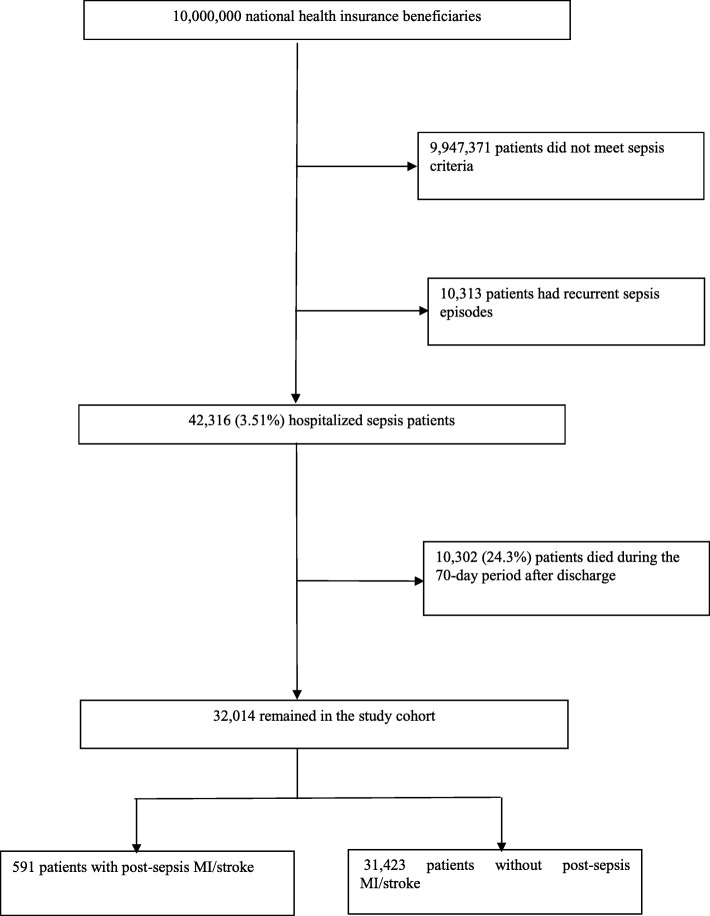
Construction of the study cohort

To ascertain the independent association between sepsis and incident myocardial infarction (MI)/stroke after sepsis hospitalization, we constructed a non-sepsis comparison cohort using the risk set sampling and propensity score matching technique. The non-sepsis comparison cohort was constructed using a two-stage procedure. In the first stage, we used the risk set sampling method to sample 100 non-sepsis patients for each sepsis case, matching on admission date, 5-year age group, sex, and quartile Charlson Comorbidity Score (0, 1–2, 3–4 and ≥ 5) [18]. In the second stage, we created a propensity score (PS) consisting of a comprehensive set of covariates associated with sepsis (Additional file 1: Table S1). We performed 1:1 PS matching using a greedy algorithm to construct the final non-sepsis comparison cohort. The cohort construction process is shown in Fig. 1.

### Identification of post-sepsis cardiovascular complications

As the primary aim was to analyze the impact of post-sepsis stroke/MI on mid- or long-term survival, the primary “exposure” in this study was incident MI/stroke after sepsis hospitalization. Our previous study found that the critical period for MI/stroke is 70 days after discharge from a sepsis hospitalization. We therefore assessed for MI/stroke within the 70-day risk period as the primary exposure of this study. Patients with stroke were identified by the presence of an ICD-9-CM diagnosis code of 433.xx or 434.xx, which have a positive predictive value (PPV) of 0.96 and 1.00, respectively, in the NHIRD database [19]. Patients with incident acute MI were identified by any primary or secondary admission diagnosis containing ICD-9-CM code of 410.xx together with a prescription for antiplatelet therapy, such as aspirin, clopidogrel, dipyridamole, and ticlopidine. Newer classes of antiplatelets were not available in Taiwan during the study period. The above search algorithm yields a PPV of 0.84 for acute MI in the NHIRD database [20]. The primary outcomes of interest were 180-day and 365-day all-cause mortality.

### Follow-up of cohort

**Fig. 2:**
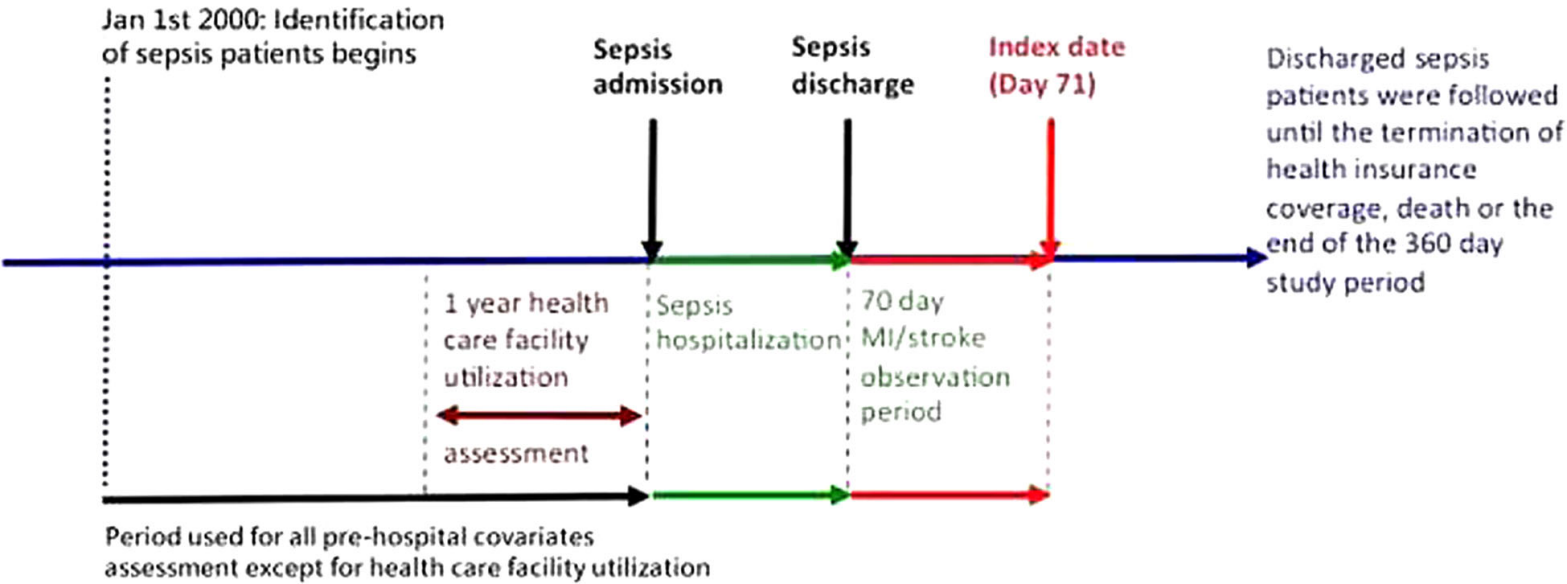
Timeline for the study design. During the study period from 2001 to 2011, patients who had post-sepsis myocardial infarction or stroke within 70 days of the last hospital discharge due to sepsis entered the cohort on the 71st day. Three outcomes were followed: the end of the 365-day study period, death, or termination of health insurance coverage

For both the primary sepsis cohort and non-sepsis comparison cohort, we anchored the 71st day after hospital discharge as the index day of cohort entry. Patients were followed up for three outcomes: death, termination of health insurance coverage, and end of study (the 365th day from the index date), whichever came first. To control for unbalanced covariates between patients with/without post-sepsis cardiovascular events, we collected information on demographics, urbanization level, insurance premium level, chronic comorbidities, and risk factors for sepsis. In order to remove the effect of covariates that could develop after incident cardiovascular events, we collected all covariate information from the beginning of the study (year 2001) to the discharge date of the index hospitalization. The level of urbanization of the cities/towns was stratified into four levels based upon a composite score obtained by calculating population density (people/km^2^), population percent of people with an educational level of college or above (%), percent of people over 65 years (%), percent of agriculture workers (%), and the number of physicians per 100,000 people. To further control for general health conditions that are not reflected by ICD-9 CM codes [21], we further collected the frequency of healthcare facility utilization in the 1-year period before sepsis admission as a proxy indicator of general health. The timeline of the study design and periods of data collection can be seen in Fig. 2.

### Data analysis

The analysis was implemented in two stages. In the primary analysis, we analyzed the impact of incident MI/stroke on the mid- or long-term outcome of sepsis survivors. In the secondary analysis, we compared the incidence and outcome of incident stoke/MI after hospital discharge between the sepsis and non-sepsis cohorts. In the primary analysis, we first compared the baseline characteristics between sepsis survivors who developed incident acute MI/stroke and those who did not develop incident acute MI/stroke. Categorical variables were presented as frequency and percentage and compared using chi-squared tests. Continuous variables were presented as mean or median and compared by non-parametric Mann-Whitney *U* tests. We then plotted the cumulative mortality curves of patients with/without post-sepsis MI/stroke. The differences in cumulative mortality between the groups were compared using the Wilcoxon signed-rank test. The survival impact of post-sepsis MI/stroke was assessed by multivariable Cox regression analyses. The proportional hazards assumption was checked by plotting curves of the log of the negative log of the survival function against log time for patients with/without post-sepsis MI/stroke. Potential confounders were determined based on the potential relevance given the literature, including the following variables: demographics, urbanization level, insurance premium, comorbidity, and healthcare service utilization. In the secondary analysis, we constructed a non-sepsis comparison cohort using the PS matching method mentioned above. To evaluate the success of the matching process, we calculated the standardized difference of matching covariates between the sepsis cohort and the non-sepsis comparison cohort. To evaluate the independent association between sepsis and post-sepsis MI/stroke, we calculated the incidence and 180-day mortality of MI/stroke among patients for the sepsis cohort and the PS-matched non-sepsis comparison cohort. We used univariate conditional logistic regression to evaluate the risk of incident MI/stroke and stratified Cox regression analysis to evaluate mortality risk. All statistical analyses were performed in SAS 9.4 (SAS Inc. Cary NC). In all analyses, a *P* value of < 0.05 was deemed significant.

## Results

### Baseline characteristics of study patients

From one million NHIRD participants, we identified 42,316 patients who were hospitalized with sepsis (Fig. 1). There were 591 events observed in the 70-day risk period after discharge, of which 486 (82.2%) were stroke, 108 (18.3%) were MI, and 3 (0.5%) were concomitant stroke and MI. The preadmission characteristics of sepsis patients with/without post-sepsis MI/stroke are summarized in Table 1. Compared with patients without MI/stroke, patients who developed MI/stroke were older, associated with lower socioeconomic status, had a higher burden of preadmission comorbidities, and had a higher frequency of healthcare service utilization.

**Table 1:** Characteristics of patients with and without post-sepsis MI/stroke

Characteristics	Patients who developed MI/stroke, *N* = 591	Patients who did not develop MI/stroke, *N* = 31,423	*P* value
Demographics			
Male sex	374 (63.28%)	18,092 (57.58%)	0.0204
Age	74.26 ± 12.45	66.11 ± 19.13	< .0001
Urbanization level, %			
Level 1: urban area	202 (34.18%)	12,058 (38.37%)	0.1386
Level 2: metro area	156 (26.40%)	8082 (25.72%)	
Level 3: suburban area	159 (26.90%)	7409 (23.58%)	
Level 4: countryside area	74 (12.52%)	3874 (12.33%)	
Insurance premium level, % (New Taiwan dollars)			
Dependent	89 (15.06%)	4443 (14.14%)	0.0044
No/poverty income level ($1–$19,999)	306 (51.78%)	14,281 (45.45%)	
Middle income level ($20,000–$39,999)	158 (26.73%)	10,107 (32.17%)	
High income level (≥ $40,000)	38 (6.43%)	2592 (8.25%)	
Preadmission comorbidity, %			
Myocardial infarction	41 (6.94%)	826 (2.63%)	< .0001
Congestive heart failure	126 (21.32%)	4691 (14.93%)	< .0001
Peripheral vascular disease	30 (5.08%)	1182 (3.76%)	0.0971
Cerebrovascular disease	320 (54.15%)	7618 (24.24%)	< .0001
Dementia	77 (13.03%)	2818 (8.97%)	0.0006
Chronic pulmonary disease	207 (35.03%)	9771 (31.1%)	0.0410
Rheumatologic disease	10 (1.69%)	407 (1.3%)	0.3993
Peptic ulcer disease	139 (23.52%)	7955 (25.32%)	0.3195
Mild liver disease	66 (11.17%)	5950 (18.94%)	< .0001
Diabetes without chronic complications	236 (39.93%)	9013 (28.68%)	< .0001
Diabetes with chronic complications	91 (15.4%)	3263 (10.38%)	< .0001
Hemiplegia or paraplegia	44 (7.45%)	1198 (3.81%)	< .0001
Renal disease	82 (13.87%)	3930 (12.51%)	0.3196
Any malignancy, including leukemia and lymphoma	46 (7.78%)	4822 (15.35%)	< .0001
Moderate or severe liver disease	8 (1.35%)	1047 (3.33%)	0.0076
Metastatic solid tumor	7 (1.18%)	1300 (4.14%)	0.0003
AIDS/HIV	2 (0.34%)	31 (0.1%)	0.0719
Alcohol/drug use	8 (1.35%)	786 (2.5%)	0.0755
Psychiatric disorder	112 (18.95%)	5341 (17%)	0.2106
Neurologic disorder	82 (13.87%)	2612 (8.31%)	< .0001
Obesity	1 (0.17%)	94 (0.3%)	1.0000
Bed-ridden status	45 (7.61%)	1226 (3.9%)	< .0001
Solid organ transplantation such as renal or heart transplantation	0 (0%)	45 (0.14%)	1.0000
Healthcare service utilization, %			
Number of OPD visits	30.96 ± 22.85	29.95 ± 22.79	0.2851
Number of emergency department visits	1.06 ± 2.33	0.85 ± 2.02	0.0116
Number of hospital admissions	1.45 ± 1.81	1.30 ± 1.88	0.0563

Summary and comparison of the demographics and underlying comorbidities of the study cohort stratified by post-sepsis MI/stroke

*MI* myocardial infarction, *AIDS/HIV* acquired immune deficiency syndrome/human immunodeficiency virus, *OPD* outpatient department

### Survival impact of post-sepsis acute MI and stroke

**Fig. 3:**
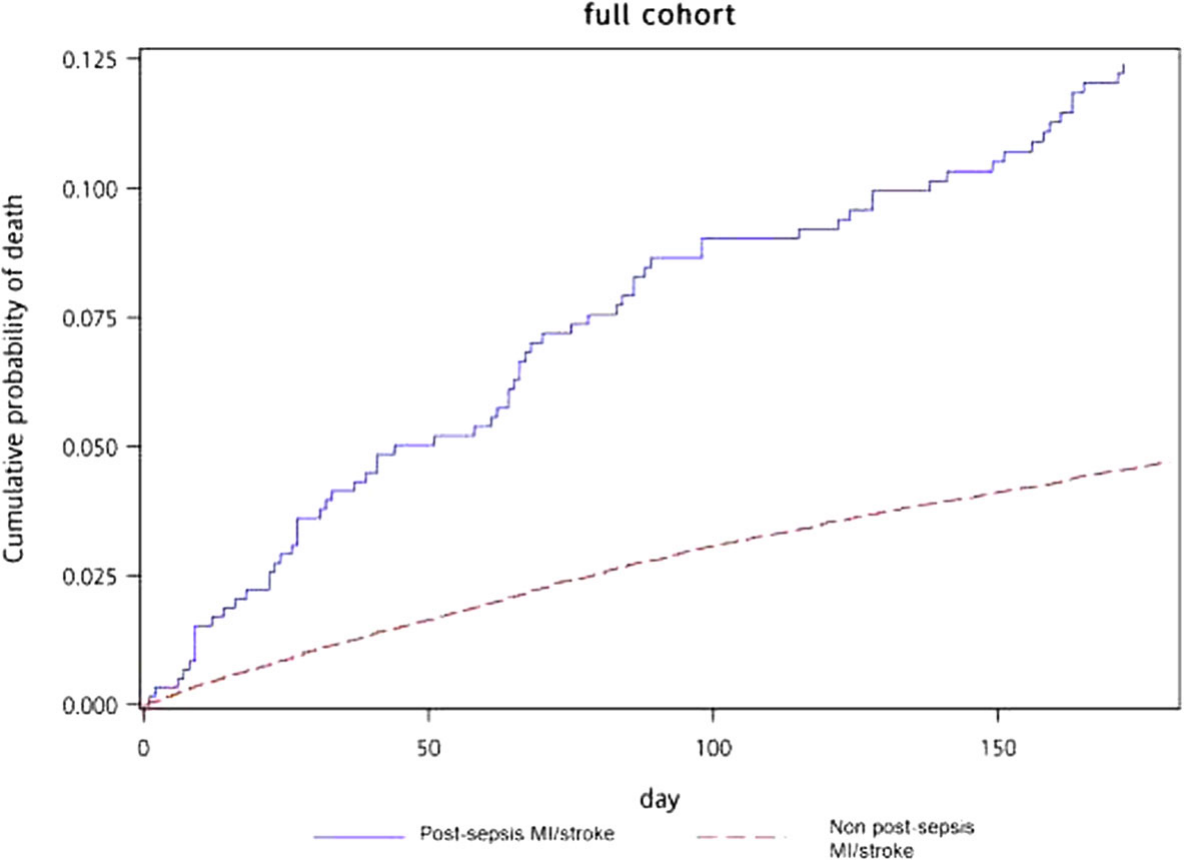
Cumulative mortality among patients with post-sepsis MI/stroke as compared with patients without post-sepsis MI/stroke in the full cohort. Visual presentation of the cumulative mortality of the study cohort. The solid line represents the cumulative mortality of the patients with post-sepsis MI/stroke, while the dotted line represents the cumulative mortality of those who did not experience post-sepsis MI/stroke. MI, myocardial infarction

The 180-day mortality for sepsis survivors without incident MI/stroke was 4.61% (1450/31,423). Patients who developed post-sepsis MI/stroke had twofold higher mortality compared to patients without post-sepsis MI/stroke. The mortality of MI/stroke-naïve sepsis survivors rose to 7.35% (2309/31,423) at 365 days. The risk of mortality remained twofold higher for patients who developed post-sepsis MI/stroke. The crude mortality results are summarized in Table 2. To evaluate the survival impact of post-sepsis MI/stroke 180 days following hospital discharge, we plotted the cumulative hazard curve in Fig. 3. We observed that patients who developed MI/stroke had a significantly higher cumulative mortality than patients without cardiovascular complications (Wilcoxon test *P* = 0.003) within the 180-day period. Adjusting for demographic variables and potential confounders (e.g., comorbidities) in the Cox proportional hazard model, we found a significant increase in 180-day mortality for patients with post-sepsis MI (hazard ratio [HR] 2.55, 95% confidence interval [CI] 1.53, 4.27), post-sepsis stroke (HR 2.19, 95% CI 1.67, 2.87), and composite post-sepsis MI/stroke (HR 2.02, 95% CI 1.65, 2.47) as compared with patients without post-sepsis MI/stroke (Table 2). At 365 days, the increased risk persisted for patients with post-sepsis MI (HR 2.10, 95% CI 1.34, 3.32), post-sepsis stroke (HR 2.02, 95% CI 1.61, 2.52), and composite post-sepsis MI/stroke (HR 2.02, 95% CI 1.66, 2.49). The full Cox model and effect estimates of the adjusted covariates are summarized in Table 3.

**Table 2:** One hundred eighty- and 365-day mortality of patients with and without post-sepsis MI/stroke

Full sepsis cohort	180 days		365 days	
	Mortality rate	Confounder-adjusted hazard ratio	Mortality rate	Confounder-adjusted hazard ratio
Patients with post-sepsis MI	13.89% (15/108)	2.55 (1.53, 4.27)	17.59% (19/108)	2.10 (1.34, 3.32)
Patients with post-sepsis stroke	11.32% (55/486)	2.19 (1.67, 2.87)	16.67% (81/486)	2.02 (1.61, 2.52)
Patients with post-sepsis MI or stroke	11.68% (69/591)	2.02 (1.65, 2.47)	16.75% (99/591)	2.02 (1.66, 2.49)
Patients without post-sepsis complications	4.61% (1450/31,423)	Reference	7.35% (2309/31,423)	Reference

Representation of the survival analysis results using Cox regression model summarizing survival impact of post-sepsis MI, post-sepsis stroke, and post-sepsis MI/stroke while accounting for potential confounders

*MI* myocardial infarction

**Table 3:** Results of multivariable analysis showing the adjusted hazard ratio associated with the 180-day and 365-day mortality of each variable

Characteristics	180-day mortality, HR (95% CI)	*P* value	365-day mortality, HR (95% CI)	*P* value
Post-sepsis MI/stroke	2.02 (1.65, 2.47)	< .0001	2.02 (1.65, 2.47)	< .0001
Demographics				
Male sex	1.38 (1.27, 1.51)	< .0001	1.38 (1.27, 1.51)	< .0001
Age	1.03 (1.02, 1.03)	< .0001	1.03 (1.02, 1.03)	< .0001
Urbanization level, %				
Level 1: urban area	1.70 (1.42, 2.03)	< .0001	1.69 (1.46, 1.95)	< .0001
Level 2: metro area	1.40 (1.16, 1.69)	0.0005	1.45 (1.25, 1.69)	< .0001
Level 3: suburban area	1.09 (0.91, 1.32)	0.3524	1.10 (0.94, 1.27)	0.2392
Level 4: countryside area	Reference	–	Reference	–
Insurance premium level, % (New Taiwan dollars)				
Dependent	Reference	–	Reference	–
No/poverty income level ($1–$19,999)	1.06 (0.94, 1.2)	0.3171	1.06 (0.94, 1.2)	0.3171
Middle income level ($20,000–$39,999)	1.25 (1.1, 1.44)	0.001	1.25 (1.1, 1.44)	0.001
High income level (≥ $40,000)	0.84 (0.68, 1.04)	0.1034	0.84 (0.68, 1.04)	0.1034
Preadmission comorbidity, %				
Myocardial infarction	1.03 (0.83, 1.28)	0.7758	1.03 (0.83, 1.28)	0.7758
Congestive heart failure	1.16 (1.04, 1.29)	0.0069	1.16 (1.04, 1.29)	0.0069
Peripheral vascular disease	0.90 (0.74, 1.1)	0.319	0.90 (0.74, 1.10)	0.319
Cerebrovascular disease	1.16 (1.06, 1.28)	0.0021	1.16 (1.06, 1.28)	0.0021
Dementia	1.47 (1.31, 1.65)	< .0001	1.47 (1.31, 1.65)	< .0001
Chronic pulmonary disease	1.11 (1.02, 1.21)	0.022	1.11 (1.02, 1.21)	0.022
Rheumatologic disease	1.27 (0.91, 1.78)	0.1602	1.27 (0.91, 1.78)	0.1602
Peptic ulcer disease	0.96 (0.88, 1.06)	0.4459	0.96 (0.88, 1.06)	0.4459
Mild liver disease	1.07 (0.95, 1.20)	0.2567	1.07 (0.95, 1.20)	0.2567
Diabetes without chronic complications	1.09 (0.99, 1.20)	0.0987	1.09 (0.99, 1.20)	0.0987
Diabetes with chronic complications	0.98 (0.85, 1.13)	0.7508	0.98 (0.85, 1.13)	0.7508
Hemiplegia or paraplegia	1.56 (0.44, 5.47)	0.4913	1.56 (0.44, 5.47)	0.4913
Renal disease	1.15 (1.02, 1.29)	0.0184	1.15 (1.02, 1.29)	0.0184
Any malignancy, including leukemia and lymphoma	1.40 (1.25, 1.57)	< .0001	1.40 (1.25, 1.57)	< .0001
Moderate or severe liver disease	1.72 (1.40, 2.10)	< .0001	1.72 (1.40, 2.10)	< .0001
Metastatic solid tumor	1.19 (0.99, 1.44)	0.0717	1.19 (0.99, 1.44)	0.0717
AIDS/HIV	2.05 (0.66, 6.39)	0.2172	2.05 (0.66, 6.39)	0.2172
Alcohol/drug use	1.08 (0.83, 1.42)	0.5569	1.08 (0.83, 1.42)	0.5569
Psychiatric disorder	0.96 (0.86, 1.07)	0.4139	0.96 (0.86, 1.07)	0.4139
Neurologic disorder	1.03 (0.90, 1.18)	0.6724	1.03 (0.90, 1.18)	0.6724
Obesity	0.75 (0.28, 2.00)	0.567	0.75 (0.28, 2.00)	0.567
Bed-ridden status	0.76 (0.22, 2.66)	0.6717	0.76 (0.22, 2.66)	0.6717
Solid organ transplantation such as renal or heart transplantation	1.26 (0.4, 3.92)	0.6922	1.26 (0.40, 3.92)	0.6922
Healthcare service utilization, %				
Number of OPD visits	1.00 (1.00, 1.00)	0.9105	1.00 (1.00, 1.00)	0.9105
Number of emergency department visits	1.02 (1.01, 1.04)	0.0002	1.02 (1.01, 1.04)	0.0002
Number of hospital admissions	1.08 (1.06, 1.10)	< .0001	1.08 (1.06, 1.10)	< .0001

Summary of the variables associated with 180-day mortality and 365-day mortality using the Cox regression model

*MI* myocardial infarction, *AIDS/HIV* acquired immune deficiency syndrome/human immunodeficiency virus, *OPD* outpatient department, *HR* hazard ratio

### Comparison between sepsis and non-sepsis cohorts

Of the 42,316 identified sepsis patients, 41,251 were matched to a non-sepsis cohort by PS matching. The standardized difference of matching covariates between sepsis and non-sepsis patients was less than 6%, indicating a successful match (Additional file 1: Table S1). We compared the incidence of post-sepsis MI/stroke between the sepsis and non-sepsis cohorts. For the sepsis cohort (*n* = 41,251), the incidence for MI, stroke, and composite MI/stroke, was 0.6%, 2.9%, and 4.2%, respectively. In contrast, for the non-sepsis cohort (*n* = 41,251), the incidence for MI, ischemic stroke, and composite MI/stroke, was 0.6%, 1.9%, and 2.7%, respectively. Compared to patients hospitalized without sepsis, patients hospitalized with sepsis had an increased risk for stroke (PS-matched OR 1.75, 95% CI 1.60–1.92) and an increased risk for composite MI/stroke (PS-matched OR 1.72, 95% CI 1.60–1.85), but not for MI alone (OR 1.01, 95% CI 0.82–1.25) (Additional file 2: Table S2). Finally, we compared mortality between the two groups of patients. For the sepsis cohort (*n* = 41,251), the 180-day mortality for MI, stroke, and composite MI/stroke was 13%, 9%, 10%, respectively. On the other hand, for the non-sepsis cohort (*n* = 41,251), the 180-day mortality for MI, stroke, and composite MI/stroke was 13%, 10%, and 11%, respectively. Cox regression showed sepsis was not associated with an increased hazard of mortality after MI, stroke, or MI/stroke (Additional file 3: Table S3).

## Discussion

Sepsis is a major public health problem. Mortality associated with sepsis may be due to post-sepsis complications. In this comparison of patients hospitalized with sepsis and patients hospitalized without sepsis matched by PS, we showed that the increased risk of cardiovascular events after sepsis hospitalization is due to sepsis per se rather than a more generalized effect of acute illness, hospitalization, or deterioration in health status. We also showed that patients who developed cardiovascular events after sepsis hospitalization have an approximately twofold increase in 180-day or 365-day mortality as compared with patients who did not sustain cardiovascular events after sepsis hospitalization. However, survival after post-hospitalization MI/stroke did not differ between sepsis and non-sepsis patients.

Several major studies have shown that infection (e.g., pneumonia, sepsis) is associated with increased risk of incident cardiovascular diseases in the short term and long term [4, 7]. Corrales-Medina et al. demonstrated that pneumonia was associated with increased short-term and long-term risk of cardiovascular diseases in two large cohorts (the Cardiovascular Health Study and the Atherosclerosis Risk in Communities Study) [7]. Wang et al. compared sepsis and non-sepsis patients in a population-based study and corroborated Corrales-Medina’s findings, showing an increased long-term risk of coronary heart disease among patients surviving sepsis [4].

In this study, we further investigated a critical yet unresolved question: whether post-sepsis MI/stroke has an independent impact on the mid- and long-term survival among sepsis survivors. Consistent with the findings by Smilowitz et al., we showed post-sepsis MI/stroke itself is an independent risk factor for mortality among sepsis survivors. Smilowitz et al. showed that the in-hospital mortality is higher among patients who had both sepsis and MI as compared with patients who had sepsis alone (adjusted odds ratio 1.24; 95% CI 1.22–1.26) [22]. However, the aforementioned study did not address the potential survivor bias [23]. We previously found the first 70 days after hospital discharge to be a period of increased susceptibility to incident cardiovascular events for sepsis patients. In addition, the mortality rate was high in the first few weeks after discharge from a sepsis hospitalization. Therefore, we indexed the cohort entry at day 70 after hospital discharge, thereby mitigating the influence of potential survivor bias or competing risk by early death. Wang et al. analyzed the impact of MI on sepsis survival, which was based on the same hospital stay. In contrast, using population-based longitudinal follow-up data, we were able to provide the evidence on the long-term outcome in patients associated with post-sepsis cardiovascular events. As shown in the current study, cardiovascular events had a major adverse impact on long-term survival of sepsis patients, suggesting a need to monitor for cardiovascular events among sepsis survivors.

The relatively low 180-day and 365-day mortality in this cohort as compared with previous reports is worth the discussion. Compared with our post-sepsis MI mortality (180-day mortality, 13.89%; 365-day mortality, 17.59%), Smilowitz et al. showed the in-hospital all-cause mortality for patients with sepsis and MI is 35.8% [22]. A similar observation was made for mortality not induced by post-sepsis MI/stroke: our 180-day (4.61%) and 356-day (7.35%) mortalities are lower than the in-hospital mortality (16.8%) reported by Smilowitz et al. Likewise, Weycker et al. and Braun et al. found post-sepsis 1-year all-cause mortality to be 51.4% and 36.1%, respectively [24, 25]. The relatively low mortality observed in our cohort could be explained by the different index dates between the current studies and previous studies. Our study design built in a lag time between hospital discharge and index date, which prevents us from gaging the high mortality of sepsis patients within this critical 70-day window. The mortality of this cohort in the 70-day period after hospital discharge was as high as 24.3%. The exclusion of patients who died within 70 days of discharge resulted in a low mortality rate in our patients.

The findings of this study support the hypothesis that the long-term mortality of sepsis is at least partially mediated through post-sepsis MI/stroke. The observed adverse survival impact of post-sepsis MI/stroke implicates the potential benefits from early preventative measures. Among the potential mediators of post-sepsis cardiovascular complications [26–31], platelets might serve as a major target for pharmacological intervention [32–36]. The protective role of long-term aspirin use in the risk of cardiovascular events is well documented [37–40], justifying future trials on its preventive role in the cardiovascular events after a sepsis episode. Such a trial may also consider including sepsis survivors without traditional cardiac risk factors. Another promising class of pharmacologic preventive agents is statins, which also have a well-established established role in the prevention of cardiovascular events. Aside from lipid-lowering properties, the pleiotropic effects of statins including anti-inflammatory and recently discovered bactericidal effects may offer added benefit to sepsis survivors [41, 42]. Definitive roles of these medications for sepsis patients awaits further investigation.

Our study has several strengths. The National Taiwan Health Insurance Database is large and comprehensive and has lent itself to many longitudinal analyses of sepsis. We used the Angus System for sepsis case identification, which is the best available algorithm for an administrative database. We used Cox regression and limited to cardiovascular events within 70 days of the sepsis event to mitigate concerns about survival time bias.

The results of this study should be interpreted in light of a few limitations. First, like other administrative databases, certain important risk factors for cardiovascular disease such as body mass index, smoking status, and alcohol consumption are lacking. Second, reverse causation may be at play given that MI or cerebrovascular events might both predispose patients to sepsis and impact the patients’ survival. To avoid the potential reverse causation, post-sepsis MI/stroke was ascertained after the hospital discharge from sepsis to establish temporality, and multivariable analyses were used to adjust for confounding effects introduced by prior myocardial infarction or cerebrovascular events. Third, with respect to our finding that there was no association between sepsis and subsequent MI, two potential causes may underlie this phenomenon. First, the comparison cohort consisted of hospitalized patients, who were already at elevated risk of MI, rather than healthy population controls. Second, the number of patients with incident MI in the sepsis cohort and the comparison cohort was only 251 and 228, respectively. As such, we did not have sufficient statistical power to detect this subtle difference.

## Conclusions

Sepsis is a major public health problem, and mortality associated with sepsis may be due to the complications that develop or accelerate after the sepsis episode. We demonstrated that the increased risk of cardiovascular events after sepsis is due to sepsis per se rather than a more generalized effect of deteriorating health status. Incident cardiovascular events after sepsis have a strong adverse impact on long-term survival. Current work may inform the need to extend sepsis care from acute stage management to the prevention of cardiovascular complications in the convalescent stage.

## Additional files

Additional file 1: **Table S1.** Characteristics of sepsis patients and propensity score-matched non-sepsis patients. Summary and comparison of the demographics and underlying comorbidities of the sepsis cohort and the non-sepsis cohort. Abbreviations: MI, myocardial infarction; AIDS/HIV, acquired immune deficiency syndrome/human immunodeficiency virus. (DOCX 17 kb)

Additional file 2: **Table S2.** Comparison of incidence (events/1000 person) of MI/stroke in sepsis and propensity score-matched non-sepsis cohort. Representation of the multivariable logistic regression analysis summarizing the association between sepsis and incidence of MI, stroke and MI/stroke while accounting for potential confounders. Abbreviations: MI, myocardial infarction; PS, propensity score; OR, odds ratio. (DOCX 15 kb)

Additional file 3: **Table S3.** Comparison of mortality of MI/stroke in sepsis and propensity score-matched non-sepsis cohorts. Representation of the survival analysis results using Cox regression model summarizing survival impact of sepsis on the mortality of MI, stroke and MI/stroke while accounting for potential confounders. Abbreviations: MI, myocardial infarction; HR, hazard ratio. (DOCX 15 kb)

Additional file 4: **Appendix 1.** Codes associated with infection. The ICD-9-CM codes utilized to identify infections. **Appendix 2.** Codes associated with organ dysfunction. The ICD-9-CM codes utilized to identify organ dysfunction. (DOCX 29 kb)

## Data Availability

The datasets generated and/or analyzed during the current study are not publicly available due to the data confidentiality requirements of the ethics committee but can be made available by the corresponding author on reasonable request and approval from the ethics committee.

## Abbreviations

ED: Emergency department
HR: Hazard ratio
ICD: International Classification of Diseases
MI: Myocardial infarction
NHIRD: National Health Insurance Research Database
