# Supplementary material for: Impact of post-sepsis cardiovascular complications on mortality in sepsis survivors: a population-based study
Source: Crit Care. 2019 Sep 2;23:293. doi: 10.1186/s13054-019-2579-2 (PMC6720410; doi:10.1186/s13054-019-2579-2)
Supplement: Supplementary file 1 — Table S1. Characteristics of sepsis patients and propensity score-matched non-sepsis patients. Summary and comparison of the demographics and underlying comorbidities of the sepsis cohort and the non-sepsis cohort. Abbreviations: MI, myocardial infarction; AIDS/HIV, acquired immune deficiency syndrome/human immunodeficiency virus. (DOCX 17 kb) [file 13054_2019_2579_MOESM1_ESM.docx]

**Additional file 1: Table S1. Characteristics of sepsis patients and propensity score-matched non-sepsis patients**

| **Characteristics** | **Sepsis patients**  **N= 41,251** | **Matched non-sepsis patients**  **N= 41,251** | **Standardized difference** |
| --- | --- | --- | --- |
| **Demographics** |  |  |  |
| Male sex | 24436 (59.24%) | 24436 (59.24%) | 0 |
| Age, year | 67.7373±17.7265 | 67.9495±17.5945 | -0.01 |
| **Comorbidity %** |  |  |  |
| Myocardial infarction | 2437 (5.91%) | 2446 (5.93%) | -0.0009 |
| Congestive heart failure | 10268 (24.89%) | 10453 (25.34%) | -0.01 |
| Peripheral vascular disease | 4718 (11.44%) | 5046 (12.23%) | -0.02 |
| Cerebrovascular disease | 15631 (37.89%) | 15058 (36.5%) | 0.03 |
| Dementia | 5460 (13.24%) | 5112 (12.39%) | 0.03 |
| Chronic pulmonary disease | 22027 (53.4%) | 22460 (54.45%) | -0.02 |
| Rheumatologic disease | 1721 (4.17%) | 2048 (4.96%) | -0.04 |
| Peptic ulcer disease | 20562 (49.85%) | 21538 (52.21%) | -0.05 |
| Mild liver disease | 16160 (39.17%) | 16877 (40.91%) | -0.04 |
| Diabetes without chronic  complications | 15495 (37.56%) | 15210 (36.87%) | 0.01 |
| Diabetes with chronic  complications | 6947 (16.84%) | 6914 (16.76%) | 0.002 |
| Hemiplegia or paraplegia | 4191 (10.16%) | 4384 (10.63%) | -0.02 |
| Renal disease | 7960 (19.3%) | 8176 (19.82%) | -0.01 |
| Any malignancy, including  leukemia and lymphoma | 8469 (20.53%) | 8060 (19.54%) | 0.02 |
| Moderate or severe liver disease | 1449 (3.51%) | 1032 (2.5%) | 0.06 |
| Metastatic solid tumor | 2041 (4.95%) | 2061 (5%) | -0.002 |
| AIDS/HIV | 63 (0.15%) | 63 (0.15%) | 0 |

Caption: *Summary and comparison of the demographics and underlying comorbidities of the sepsis cohort and the non-sepsis cohort. Abbreviations: MI, myocardial infarction; AIDS/HIV, acquired immune deficiency syndrome/human immunodeficiency virus*
