# Supplementary material for: Impact of post-sepsis cardiovascular complications on mortality in sepsis survivors: a population-based study
Source: Crit Care. 2019 Sep 2;23:293. doi: 10.1186/s13054-019-2579-2 (PMC6720410; doi:10.1186/s13054-019-2579-2)
Supplement: Supplementary file 2 — Table S2. Comparison of incidence (events/1000 person) of MI/stroke in sepsis and propensity score-matched non-sepsis cohort. Representation of the multivariable logistic regression analysis summarizing the association between sepsis and incidence of MI, stroke and MI/stroke while accounting for potential confounders. Abbreviations: MI, myocardial infarction; PS, propensity score; OR, odds ratio. (DOCX 15 kb) [file 13054_2019_2579_MOESM2_ESM.docx]

**Additional file 2: Table S2. Comparison of incidence (events/1000 persons) of MI/stroke in sepsis and propensity score-matched non-sepsis cohorts**

| **Incidence of MI in sepsis cohort** | **Incidence of MI in non-sepsis cohort** | **PS-matched OR (95% confidence interval)** |
| --- | --- | --- |
| 251/41251 (0.6%) | 228/41251 (0.6%) | 1.01 (0.82,1.25) |
| **Incidence of stroke in sepsis cohort** | **Incidence of stroke in non-sepsis cohort** | **PS-matched OR (95% confidence interval)** |
| 1178/41251 (2.9%) | 771/41251 (1.9%) | 1.75 (1.60,1.92) |
| **Incidence of composite MI/stroke in sepsis cohort** | **Incidence of composite MI/stroke in non-sepsis cohort** | **PS-matched OR (95% confidence interval)** |
| 1725/41251 (4.2%) | 1132 /41251 (2.7%) | 1.72 (1.60,1.85) |

Caption: *Representation of the multivariable logistic regression analysis summarizing the association between sepsis and incidence of MI, stroke and MI/stroke while accounting for potential confounders. Abbreviations: MI, myocardial infarction; PS, propensity score; OR, odds ratio*
