# Supplementary material for: Impact of post-sepsis cardiovascular complications on mortality in sepsis survivors: a population-based study
Source: Crit Care. 2019 Sep 2;23:293. doi: 10.1186/s13054-019-2579-2 (PMC6720410; doi:10.1186/s13054-019-2579-2)
Supplement: Supplementary file 3 — Table S3. Comparison of mortality of MI/stroke in sepsis and propensity score-matched non-sepsis cohorts. Representation of the survival analysis results using Cox regression model summarizing survival impact of sepsis on the mortality of MI, stroke and MI/stroke while accounting for potential confounders. Abbreviations: MI, myocardial infarction; HR, hazard ratio. (DOCX 15 kb) [file 13054_2019_2579_MOESM3_ESM.docx]

**Additional fi8le 3: Table S3. Comparison of 180-day mortality of MI/stroke in sepsis and propensity score-matched non-sepsis cohorts**

| **Mortality of sepsis patients with MI** | **Mortality of non-sepsis patients with MI** | **HR (95% confidence interval)** |
| --- | --- | --- |
| 22 / 172 (13%) | 14 / 109 (13%) | 0.99 (0.82,1.18) |
| **Mortality of sepsis patients with stroke (ICD-9 433-438)** | **Mortality of non-sepsis patients with stroke** | **HR (95% confidence interval)** |
| 732 / 796 (9%) | 32 / 335 (10%) | 0.92 (0.82,1.02) |
| **Mortality of sepsis patients with composite MI/stroke** | **Mortality of non-sepsis patients with composite MI/stroke** | **HR (95% confidence interval)** |
| 122 / 1171 (10%) | 54 / 507 (11%) | 0.95 (0.87,1.02) |

Caption: *Representation of the survival analysis results using Cox regression model summarizing survival impact of sepsis on the mortality of MI, stroke and MI/stroke while accounting for potential confounders. Abbreviations: MI, myocardial infarction;* *HR, hazard ratio*
