# Supplementary material for: Impact of post-sepsis cardiovascular complications on mortality in sepsis survivors: a population-based study
Source: Crit Care. 2019 Sep 2;23:293. doi: 10.1186/s13054-019-2579-2 (PMC6720410; doi:10.1186/s13054-019-2579-2)
Supplement: Supplementary file 4 — Appendix 1. Codes associated with infection. The ICD-9-CM codes utilized to identify infections. Appendix 2. Codes associated with organ dysfunction. The ICD-9-CM codes utilized to identify organ dysfunction. (DOCX 29 kb) [file 13054_2019_2579_MOESM4_ESM.docx]

**Additional file 4**

**Appendix 1: Codes associated with infection**

**Codes associated with infection**

**Gastrointestinal infection**

001, Cholera;

002, Typhoid/paratyphoid fever;

003, Other salmonella infection;

004, Shigellosis;

005 Other food poisoning;

008, Intestinal infection not otherwise classified;

009, Ill-defined intestinal infection;

562.01, Diverticulitis of small intestine without hemorrhage;

562.03, Diverticulitis of small intestine with hemorrhage;

562.11, Diverticulitis of colon without hemorrhage;

562.13, Diverticulitis of colon with hemorrhage;

**Tuberculosis**

010, Primary tuberculosis infection;

011 Pulmonary tuberculosis;

012, Other respiratory tuberculosis;

013, Central nervous system tuberculosis;

014, Intestinal tuberculosis; 015, Tuberculosis of bone and joint; 016, Genitourinary tuberculosis;

017, Tuberculosis not otherwise classified;

018, Miliary tuberculosis;

**Zoonosis**

020, Plague;

021, Tularemia;

022, Anthrax;

023, Brucellosis;

024, Glanders;

025, Melioidosis;

026, Rat-bite fever;

027, other bacterial zoonoses;

**Other mycobacterial disease**

030, Leprosy;

031, Other mycobacterial disease;

**Syphilis**

090, Congenital syphilis;

091, Early symptomatic syphilis;

092, Early syphilis latent;

093, Cardiovascular syphilis;

094, Neurosyphilis;

095, Other late symptomatic syphilis;

096, Late syphilis latent;

097, Other and unspecified syphilis;

102, Yaws;

103, Pinta;

104, Other spirochetal infection;

**037, Tetanus;**

**Other bacterial diseases**

040, Other bacterial diseases;

041, Bacterial infection in other diseases not otherwise specified;

**Systematic fungal infection**

110, Dermatophytosis;

111, Dermatomycosis not otherwise classified or specified;

112, Candidiasis;

114, Coccidioidomycosis;

115, Histoplasmosis;

116, Blastomycotic infection;

117, Other mycoses;

118, Opportunistic mycoses;

117.9 Disseminated fungal infection

112.5 Dissemintaed candidal infection

112.81 Disseminated fungal endocarditis

039, Actinomycotic infections;

**CNS infection**

320, Bacterial meningitis;

322, Meningitis, unspecified;

324, Central nervous system abscess;

325, Phlebitis of intracranial sinus;

036, Meningococcal infection;

**Cardiovascular infection**

420, Acute pericarditis;

421, Acute or subacute endocarditis;

451, Thrombophlebitis;

**Upper respiratory tract infection**

461, Acute sinusitis;

462, Acute pharyngitis;

463, Acute tonsillitis;

464, Acute laryngitis/ tracheitis;

465, Acute upper respiratory infection of multiple sites/not otherwise specified;

101, Vincent’s angina;

034, Streptococcal throat/scarlet fever;

032, Diphtheria;

**Lower respiratory tract infection**

481, Pneumococcal pneumonia;

482, Other bacterial pneumonia;

485, Bronchopneumonia with organism not otherwise specified;

486, Pneumonia, organism not otherwise specified;

491.21, Acute exacerbation of obstructive chronic bronchitis;

494 Bronchiectasis;

510, Empyema;

513, Lung/mediastinum abscess;

033, Whooping cough;

484 Pneumonia classified in elsewhere

483 Pneumonia by other pathogens

**Intra-abdominal infection**

540, Acute appendicitis;

541, Appendicitis not otherwise specified;

542, Other appendicitis;

566, Anal and rectal abscess;

567, Peritonitis;

569.5, Intestinal abscess;

569.83, Perforation of intestine;

572.0, Abscess of liver;

**Biliary tract infection**

572.1, Portal pyremia;

575.0, Acute cholecystitis;

**Genitourinary tract infection**

590, Kidney infection;

597, Urethritis/urethral syndrome;

599.0, Urinary tract infection not otherwise specified;

601, Prostatic inflammation;

098, Gonococcal infections;

**Gynecological infection**

614, Female pelvic inflammation disease;

615, Uterine inflammatory disease;

616, Other female genital inflammation; 681, Cellulitis, finger/ toe;

098, Gonococcal infections;

**Skin and appendix structure infection**

682, Other cellulitis or abscess;

683, Acute lymphadenitis;

686, Other local skin infection;

035, Erysipelas;

**Musculoskeletal infection**

711.0, Pyogenic arthritis;

730, Osteomyelitis; 790.7, Bacteremia;

996.6, Infection or inflammation of device/graft;

**Post-operative complication**

998.5, Postoperative infection;

**Nosocomial infection**

999.3, Infectious complication of medical care not otherwise classified.

**Septicemia**

038.0 Streptococcal septicemia

038.1 Staphylococcal septicemia

038.2 Pneumococcal septicemia [Streptococcus pneumoniae septicemia]

038.3 Septicemia due to anaerobes

Septicemia due to Bacteroides

Excludes: gas gangrene (040.0), that due to anaerobic streptococci (038.0)

038.4 Septicemia due to other gram-negative organisms

038.40 Gram-negative organism, unspecified

Gram-negative septicemia NOS

038.41 Hemophilus influenzae

038.42 Escherichia coli

038.43 Pseudomonas

038.44 Serratia

038.8 Other specified septicemias, excluding septicemia due to anthrax (022.3), gonococcal (098.89), herpetic (054.5), meningococcal (036.2), septicemic plague (020.2)

- 1. Unspecified septicemia

Septicemia NOS

Excludes: bacteremia NOS (790.7)

995.92 Severe sepsis

790.7 Bacteremia

**Appendix 2: Codes associated with organ dysfunction**

**Cardiovascular dysfunction/Shock**

Use of vasopressor (dopamine, norepinephrine, and epinephrine) and (Shock 785.5 or hypotension 458)

**Acute respiratory failure**

Mechanical ventilation 96.7 or use of ventilator 57001B,57002B, 57023B, 57029C

**Central nervous system dysfunction**

Neurologic Encephalopathy 348.3

Transient organic psychosis 293

Anoxic brain damage 348.1

**Hematologic system dysfunction**

Hematologic Secondary thrombocytopenia 287.4

Thrombocytopenia, unspecified 287.5

Other/unspecified coagulation defect 286.9

Defibrination syndrome 286.6

**Hepatic system dysfunction**

Hepatic Acute and subacute necrosis of liver 570

Hepatic encephalopathy 572.2

Hepatorenal syndrome 572,4

Other squeal of chronic liver disease 572.8

Hepatic infarction 573.4, 573.8

Liver replaced by transplant V42.7

**Renal system dysfunction**

Acute renal failure 584 or Use of CVVH (58014C)

**Metabolic system dysfunction (Hyperglycemic crisis)**

Diabetic ketoscidosis 250.1

Hyperosmolar hyperglycemic state 250.2
